# Age-dependent changes in the neural correlates of force modulation: An fMRI study

**DOI:** 10.1016/j.neurobiolaging.2007.04.017

**Published:** 2008-09

**Authors:** Nick S. Ward, Orlando B.C. Swayne, Jennifer M. Newton

**Affiliations:** a Wellcome Department of Imaging Neuroscience, Institute of Neurology, University College London, 12 Queen Square, London WC1N 3BG, UK; b Department of Brain Repair and Rehabilitation, Institute of Neurology, University College, London, UK; c Sobell Department of Motor Neuroscience and Movement Disorders, Institute of Neurology, University College, London, UK

**Keywords:** Motor system, fMRI, Aging, BOLD, Hand grip, Dominant, Non-dominant

## Abstract

Functional imaging studies in humans have demonstrated widespread age-related changes in cortical motor networks. However, the relative contribution of cortical regions during motor performance varies not only with age but with task parameters. In this study, we investigated whether motor system activity during a task involving increasingly forceful hand grips was influenced by age. Forty right-handed volunteers underwent functional magnetic brain imaging whilst performing repetitive isometric hand grips with either hand in separate sessions. We found no age-related changes in the average size and shape of the task-related blood oxygen level dependent (BOLD) signal in contralateral primary motor cortex (M1), but did observe reduced ipsilateral M1 deactivation in older subjects (both hands). Furthermore, task-related activity co-varied positively with force output in a number of brain regions, but was less prominent with advancing age in contralateral M1, cingulate sulcus (both hands), sensory and premotor cortices (right hand). These results indicate that a reduced ability to modulate activity in appropriate motor networks when required may contribute to age-related decline in motor performance.

## 1 Introduction

Aging is associated with alterations in musculoskeletal architecture (Dutta et al., 1997; Lexell, 1995, 1997), peripheral and central nerve conduction (Dorfman and Bosley, 1979), proprioception (Kaplan et al., 1985) and neuromuscular coupling (Delbono, 2003; Lexell, 1997). In the central nervous system, the size of neurons (Haug and Eggers, 1991), number of synapses (Haug and Eggers, 1991), integrity of white matter (Madden et al., 2004), volume of grey matter (Good et al., 2001) and neurotransmitter levels (Volkow et al., 1998; Wenk et al., 1989) have all been reported to decrease with advancing age. These neurodegenerative and neurochemical changes are thought to underlie a decline in cognitive and motor function (Hackel et al., 1992; Jebsen et al., 1969; Kolb et al., 1998; Potvin et al., 1980).

Several studies have examined the neural correlates of aging using functional imaging. In general, task-related activation appears to be focused and lateralised in younger subjects but more diffuse and bilateral with advancing age (Cabeza, 2001). Studies concentrating on the motor system have used a variety of tasks ranging from simple to complex (Calautti et al., 2001; Hesselmann et al., 2001; Heuninckx et al., 2005; Hutchinson et al., 2002; Mattay et al., 2002; Naccarato et al., 2006; Riecker et al., 2006; Rowe et al., 2006; Tekes et al., 2005; Ward and Frackowiak, 2003; Wu and Hallett, 2005). Age-related differences are greater when complex tasks are used, but usually in non-motor brain regions, which may reflect increased cognitive monitoring of performance (Heuninckx et al., 2005). The effects of advancing age on activity in primary motor cortex (M1) are less clear, with reports of decreased (D’Esposito et al., 1999; Hesselmann et al., 2001; Hutchinson et al., 2002; Riecker et al., 2006; Tekes et al., 2005; Wu and Hallett, 2005) increased (Mattay et al., 2002; Naccarato et al., 2006), or unchanged (Calautti et al., 2001; Heuninckx et al., 2005; Ward and Frackowiak, 2003) activation in contralateral M1 in older subjects. Two studies have reported less deactivation in ipsilateral M1 with increasing age (Naccarato et al., 2006; Ward and Frackowiak, 2003). The disparities in these findings may be due to differences in the motor task and the method of analysis used (D’Esposito et al., 1999; Ward, 2006). Experiments using transcranial magnetic stimulation (TMS) however, have consistently demonstrated reduced excitability of the corticospinal pathway (Oliviero et al., 2006; Pitcher et al., 2003; Sale and Semmler, 2005) and decreased excitability of intracortical inhibitory circuits (Oliviero et al., 2006; Peinemann et al., 2001; Sale and Semmler, 2005) in older subjects.

We therefore carried out a functional magnetic resonance imaging (fMRI) experiment to specifically look for age-related changes in the task-related behaviour of motor and motor-related cortical regions. Subjects were asked to perform a simple isometric hand grip task with parametric modulation of force output. We used a sparse event-related design with target forces set as a proportion of each subjects own maximum grip force in order to ensure that our results would not be affected by differences in task performance or perceived task difficulty. Furthermore, we used random effects model to analyse the data in order to minimise the potential effect of a reduced blood oxygen level dependent (BOLD) signal to noise ratio in older subjects (D’Esposito et al., 1999).

Our first aim was to examine for age-related changes in the average magnitude and shape of BOLD responses during hand grip. Secondly, in view of the neurophysiological findings suggesting a reduced ability to engage the corticospinal system by motor commands in older subjects (Pitcher et al., 2003; Sale and Semmler, 2005), we also looked for age-related differences in brain regions involved in the modulation of force production. Previous studies in healthy humans have shown increasing activity in brain regions contributing to corticospinal projections with increasing force production (Dettmers et al., 1995; Thickbroom et al., 1998; Ward and Frackowiak, 2003). We predicted that the force-related increase in brain activity would be less prominent in older subjects, indicating a reduced ability to change brain activity in response to changing motor task parameters.

## 2 Methods

### 2.1 Subjects

Forty healthy volunteers (age range 21–75 years, mean 48.9 years, S.D. 16.9 years) comprising 22 male subjects and 18 female subjects, participated in the study. All subjects were right handed according to the Edinburgh handedness scale (Oldfield, 1971). They reported no history of neurological illness, psychiatric history, vascular disease or hypertension. Subjects were not taking regular medication. Full written consent was obtained from all subjects in accordance with the Declaration of Helsinki. The study was approved by the Joint Ethics Committee of the Institute of Neurology, UCL and National Hospital for Neurology and Neurosurgery, UCL Hospitals NHS Foundation Trust, London.

### 2.2 Behavioural evaluation

Maximum grip strength with each hand was measured for each subject using a Jamar hydraulic hand dynamometer (Fabrication Enterprises, Inc., NY, USA).

### 2.3 fMRI scanning

#### 2.3.1 Motor paradigm

All subjects underwent two consecutive scanning sessions, one using the dominant right hand, and one using the non-dominant left hand. The order of these sessions was randomised and counterbalanced across subjects. During scanning, all subjects performed a series of dynamic isometric hand grips using a MRI-compatible manipulandum as previously described (Ward and Frackowiak, 2003). Continuous visual feedback about the force exerted was provided. Prior to scanning, but whilst lying in the scanner, subjects were asked to grip the manipulandum using maximum force to generate a maximum voluntary contraction (MVC) for each hand. A single scanning session comprised 30 visually cued hand grips interspersed with 30 null events in a randomised and counterbalanced order (SOA = 5.72 s, scanning time 6 min 14 s). The onset and target force of each single hand grip was visually cued. The target force was varied such that ten grips at each of 15%, 30% and 45% of MVC were performed (30 in total) in a random order. Subjects were instructed to use the visual target as a guide to the level of force production, but were not specifically asked to be accurate in order to avoid prolonged hand grips. All subjects practised the motor task in two 3 min blocks: once outside the scanner and for a second time in the scanner before scanning started. To look for bilateral movements during scanning subjects held identical hand grip manipulanda in both hands while carrying out the task unimanually.

#### 2.3.2 Data acquisition

A 3T Siemens ALLEGRA system (Siemens, Erlangen, Germany) was used to acquire both T_1_-weighted anatomical images and T_2_^*^-weighted MRI transverse echo-planar images (EPI) (64 mm × 64 mm, 3 mm × 3 mm pixels, TE = 30 ms) with BOLD contrast. Each echoplanar image comprised forty-eight 2 mm thick contiguous axial slices taken every 3 mm, positioned to cover the whole cerebrum, with an effective repetition time (TR) of 3.12 s per volume. In total, 120 volumes were acquired during each scanning session. The first six volumes were discarded to allow for T_1_ equilibration effects.

#### 2.3.3 Data preprocessing

Imaging data were analysed using Statistical Parametric Mapping (SPM5, Wellcome Department of Imaging Neuroscience, http://www.fil.ion.ucl.ac.uk/spm/) implemented in Matlab 6 (The Mathworks Inc., USA) (Friston et al., 1995b; Worsley and Friston, 1995). All volumes were realigned and slice-time corrected. No subject moved more than 2 mm in any direction, but some of this movement was task-related. In order to remove some of this unwanted movement-related variance without removing variance attributable to the motor task, realigned images were processed using the ‘unwarp’ toolbox in SPM5 (Andersson et al., 2001) which is predicated on the assumption that susceptibility-by-movement interaction is responsible for a sizeable part of residual movement-related variance. Given the observed variance (after realignment) and the realignment parameters, estimates of how deformations changed with subject movement were made, which were subsequently used to minimise movement-related variance.

The resulting volumes were then normalised to a standard EPI template based on the Montreal Neurological Institute (MNI) reference brain in Talairach space (Talairach and Tournaux, 1998) and resampled to 3 mm × 3 mm × 3 mm voxels. All normalised images were then smoothed with an isotropic 8 mm full-width half-maximum Gaussian kernel to account for intersubject anatomical differences and allow valid statistical inference according to Gaussian random field theory (Friston et al., 1995a). The time series in each voxel were high pass filtered at 1/128 Hz to remove low frequency confounds and scaled to a grand mean of 100 over voxels and scans within each session.

#### 2.3.4 Statistical analysis

Statistical analysis was performed in two stages. In the first stage, data from the right and left hand of each subject was analysed separately using a single subject single session fixed effects model. All hand grips were defined as a single event type and modelled as delta functions (grip covariate). A second covariate (force covariate) comprised a delta function scaled by the actual peak force exerted for each hand grip. The force covariate was mean corrected and orthogonalised with respect to the grip covariate. Both covariates were convolved with a canonical synthetic haemodynamic response function (HRF) together with its temporal and dispersion derivatives (Friston et al., 1998a) and used in a general linear model (Friston et al., 1995b) together with a single covariate representing the mean (constant) term over scans. The canonical HRF represents a typical BOLD response derived from a principal component analysis of data reported by Friston et al. (1998b). The temporal derivative is approximated by the orthogonalised finite difference between canonical HRF of peak delay of 7 s versus 6 s, whereas the dispersion derivative is approximated by the orthogonalised finite difference between canonical HRF of peak dispersions of 1 versus 1.01 (Friston et al., 1998a). Thus for each subject, voxel-wise parameter estimates for each covariate resulting from the least mean squares fit of the model to the data were calculated. The parameter estimates (or betas) for the grip covariate reflect the magnitude of increase in the BOLD signal during all hand grips compared to rest (*B*_G_). Positive parameter estimates of the temporal derivative (*B*_T_) result when the evoked haemodynamic response peak occurs earlier than the canonical haemodynamic response function. Positive parameter estimates of the dispersion derivative (*B*_D_) result when evoked haemodynamic response has a shorter duration compared to the canonical haemodynamic response function. Parameter estimates for the force covariate (*B*_F_) represent the partial correlation coefficient of BOLD signal plotted against hand grip force, i.e. the degree to which BOLD signal changes linearly with hand grips of different force (Buchel et al., 1998). The statistical parametric maps of the *t* statistic (SPM{*t*}) resulting from linear contrasts of each covariate (Friston et al., 1995b) were generated and stored as separate images for each subject.

The data for the second stage of analysis comprised the pooled parameter estimates for each covariate across all subjects. Contrast images for each subject were entered into a one sample *t*-test for each covariate of interest. The SPM{*t*}s were thresholded at *P* < 0.05, corrected for multiple comparisons across whole brain.

After characterizing the average group effects, we were interested in examining for the influence of age on the parameter estimates *B*_G_, *B*_T_, *B*_D_ and *B*_F_. Thus, we performed simple linear regression analyses within SPM5, in which the two orthogonal covariates were: (i) contrast images for each subject for the effect of interest (*B*_G_, *B*_T_, *B*_D_ or *B*_F_) and (ii) a single value representing age^2^ for each subject (mean corrected and normalized across the group). We hypothesized a priori that we would find non-linear changes in activation in keeping with previous behavioural (Smith et al., 1999) and imaging (Ward and Frackowiak, 2003) data and so chose to use age^2^ rather than age as the second covariate. SPM{*t*}s representing brain regions in which there is a linear relationship between the relevant parameter estimates and age^2^ were generated. The height threshold was set at *P* < 0.001, uncorrected, for multiple comparisons across whole brain, and the extent (or cluster) threshold set at *P* < 0.05, corrected for multiple comparisons across whole brain. For significant voxels the correlation coefficient for the plot of parameter estimate against age^2^ was also calculated to illustrate the relationship.

All SPM{*t*}s were transformed to the unit normal *Z*-distribution to create a statistical parametric map (SPM{*Z*}). All *t*-tests carried out within SPM were one tailed.

Anatomical identification was carefully performed by superimposing the maxima of activation foci both on the MNI brain and on the normalised structural images of each subject, and labelling with the aid of the atlas of Duvernoy (1991).

## 3 Results

### 3.1 Behavioural results

The mean MVC for each hand was calculated (right hand, mean MVC = 43.5 kg, S.D. = 5.5 kg; left hand, mean MVC = 39.8 kg, S.D. = 4.3 kg). There was no significant correlation between age or age^2^ and peak MVC for either hand. There were no significant gender-related differences in MVC and no interaction between age or age^2^ and gender.

### 3.2 Imaging result: group

#### 3.2.1 Main effects of hand grip

**Supplementary Fig. 1:**
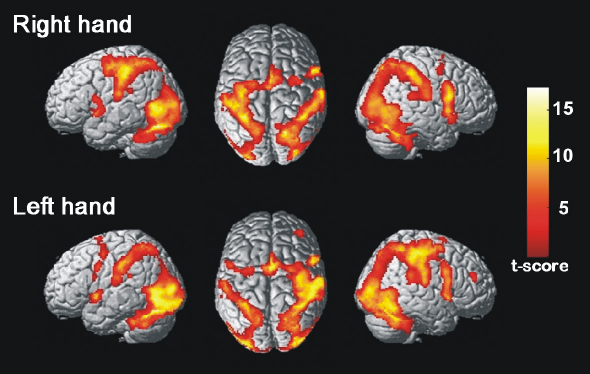
SPM {*Z*}s representing the main effect of hand grip as detected using a canonical haemodynamic response function. Results are rendered onto a canonical 3D brain. The top row of images represent results for right hand use, and the bottom row represent left hand use. The first column of images (from left to right) are seen from the left side of the brain, the middle row are seen from above (front of brain uppermost) and the third column are seen from the right. Voxels are significant at *P* < 0.001 (corrected) for the purposes of display.

**Fig. 1:**
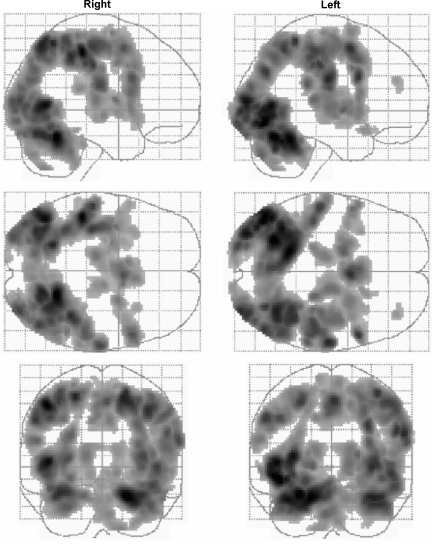
SPM {*Z*}s representing the main effect of hand grip as detected using a canonical haemodynamic response function. Results are displayed on ‘glass brains’ in two columns representing results from using the right and left hands, respectively. The glass brains are shown from the right side (top image), from above (middle image), and from below (bottom image). Voxels are significant at *P* < 0.001 (corrected) for the purposes of display.

The main effects of hand grip were consistent with previous reports using this paradigm (Ward and Frackowiak, 2003). Activations were seen in a network of regions, which was similar for right and left hands (Fig. 1 and Supplementary Fig. 1). The most lateralised activations were in contralateral sensorimotor cortex and ipsilateral superior cerebellum. Other activations were bilaterally distributed, including dorsolateral premotor cortex (PMd) and ventrolateral premotor cortex (PMv), supplementary motor area (SMA), cingulate motor areas, inferior parietal cortex and intraparietal sulcus, insula cortex, visual cortices, cerebellar vermis, and both inferior and superior cerebellar hemispheres.

#### 3.2.2 Temporal and dispersion derivatives

Earlier responses (positive parameter estimates for the temporal derivative) were seen in the pulvinar bilaterally for both the right and left hand task.

Later responses (negative parameter estimates for the temporal derivative) were observed in the left cerebellum (crus I) and right parietal cortex for both the right and left hand task. The right parietal clusters were centred on the intraparietal sulcus but extended into both superior and inferior parietal cortex.

**Fig. 2:**
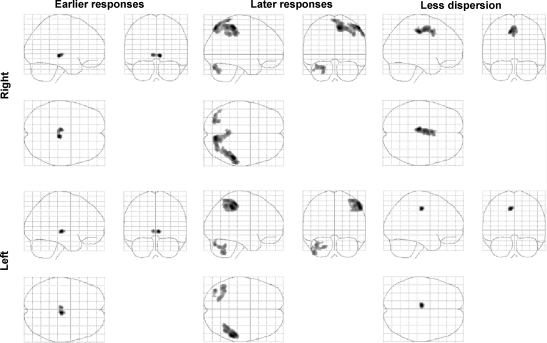
SPM {*Z*}s representing variations from the ‘canonical’ main effects of hand grip detected using temporal and dispersion derivatives of the canonical haemodynamic response function. Each result is displayed on three ‘glass brains’ shown from the right side (top left image of each group), from behind (top right image of each group), and from above (bottom left image of each group). Results are displayed in two rows representing right and left hand, respectively. Voxels are significant at *P* < 0.05 (corrected).

There were no regions in which the neural response was more dispersed (negative parameter estimates for the dispersion derivative). Less dispersion (positive parameter estimates for the dispersion derivative), i.e. shorter duration of haemodynamic response, was observed in left posterior superior cingulate sulcus for both the right and left hand task (Table 1; Fig. 2)

#### 3.2.3 Force-related changes

Regions in which the magnitude of task-related signal increased linearly with increasing hand grip force were seen in contralateral anterior M1 (BA 4a) and superior cingulate sulcus, ipsilateral cerebellum (lobule VI), and primary visual cortex for both the right and left hand task (Table 2). At a lower threshold (*P* < 0.001, uncorrected) the cluster centred on contralateral M1 (BA 4a) was found to extend in the rostral-caudal direction from *y* = −38 to −7 for the right hand and from *y* = −34 to −2 for the left hand. In the ventral–dorsal direction, the cluster extended from *z* = 38 to 74 for the right hand and from *z* = 32 to 68 for the left hand. Both these clusters additionally encompassed contralateral primary sensory cortex (S1), posterior M1 (BA 4p) and caudal dorsolateral premotor cortex.

There was a trend for decreasing magnitude of signal change with increasing grip force with the right hand in left (contralateral) ventrolateral premotor cortex (*x* = −56, *y* = 14, *z* = 36, *Z*-score = 4.19).

### 3.3 Imaging results: age-related changes

#### 3.3.1 Age-related changes in the main effects of hand grip

**Fig. 3:**
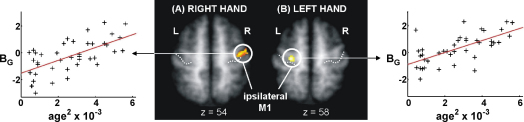
The effects of age on ipsilateral primary motor cortex (M1) activation during (A) right hand and (B) left hand use are shown. The parameter estimates for the main effects of hand grip (*B*_G_) in ipsilateral M1 are plotted against age^2^. The significant clusters (*P* < 0.05, corrected) are overlaid onto the mean normalised T_1_-weighted structural image obtained from all subjects. Peak co-ordinates, *Z*-scores and correlation coefficients are given in Table 3.

There were no negative correlations between task-related changes in signal and increasing age^2^, but positive correlations (i.e. greater task-related signal change with increasing age^2^) were observed in a number of brain regions, more so for the left hand task than right hand task (Table 3). There were no age-related changes in contralateral M1, but a positive correlation between task-related signal and increasing age^2^ was found in ipsilateral M1 for both the right and left hand task. In younger subjects, task-related signal change in ipsilateral M1 was reduced compared to rest as previously described (Newton et al., 2005), but this reduction was less marked in older subjects (Fig. 3). In some older subjects, *B*_G_ (the parameter estimate representing the average signal change for all hand grips compared to rest) was positive, indicating greater activity during hand grip compared to rest. Age-related increases in task-related signal were also seen in the putamen bilaterally for both right and left hand task. Further age-related increases were noted in dorsolateral premotor cortex bilaterally and in left (ipsilateral) intraparietal sulcus, though only for the left hand task.

#### 3.3.2 Age-related changes in the temporal and dispersion derivatives

No brain regions exhibited earlier or later haemodynamic responses as a function of age^2^ with either hand.

Task-related responses were more dispersed (i.e. longer) in older subjects in bilateral intraparietal sulcus and bilateral cerebellum (lobule VI) during the left hand task (Table 4). Right hand task responses were more dispersed in older subjects in right intraparietal sulcus only.

#### 3.3.3 Age-related changes in response to force modulation

**Fig. 4:**
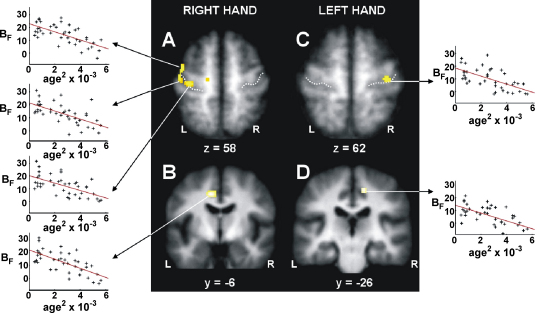
The effects of age on force modulation properties are shown. Results are shown for both right (A and B) and left (C and D) hand use. Parameter estimates for the force covariate, *B*_F_ (which represents the partial correlation coefficient between BOLD signal and hand grip force), are plotted against age^2^ for (A) contralateral primary motor cortex, primary sensory cortex and dorsolateral premotor cortex; (B) contralateral cingulate sulcus; (C) contralateral primary motor cortex; (D) contralateral cingulate sulcus. The significant clusters (*P* < 0.05, corrected) are overlaid onto the mean normalised T_1_-weighted structural image obtained from all subjects. Peak co-ordinates, *Z*-scores and correlation coefficients are given in Table 5.

The parameter estimate for the force covariate (*B*_F_) represents the partial correlation coefficient of BOLD signal plotted against hand grip force for each subject. When the right hand was used, a negative correlation between *B*_F_ and age^2^ was seen in contralateral primary sensory cortex, primary motor cortex, dorsolateral premotor cortex and anterior cingulate sulcus. In other words, the degree to which BOLD signal consistently increases with hand grips of increasing force diminished with increasing age in these brain regions. When the left hand was used, the negative correlation was observed only in contralateral primary motor cortex and posterior cingulate sulcus (Fig. 4).

**Fig. 5:**
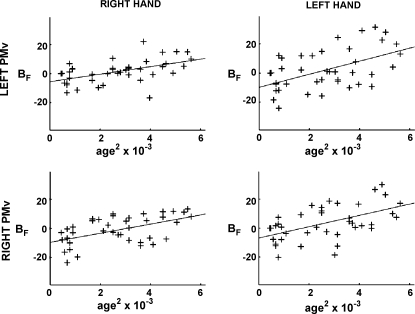
The effects of age on force modulation properties of the right (upper row) and left (lower row) ventrolateral premotor cortex (PMv) are shown. Results are shown for both right (left column) and left (right column) hand use. Parameter estimates for the force covariate, *B*_F_ (which represents the partial correlation coefficient between BOLD signal and hand grip force), are plotted against age^2^. Peak co-ordinates, *Z*-scores and correlation coefficients are given in the results section.

No positive correlations between *B*_F_ and age^2^ were observed at the statistical threshold used. However, we have previously reported that brain activity in ventral premotor cortex/BA44 bilaterally co-varies positively with peak grip force in older subjects more than younger subjects (Ward and Frackowiak, 2003), and also in stroke patients with greater damage to corticospinal tract (Ward et al., 2007). Furthermore, in the current study we observed that with increasing age there was an overall trend towards greater brain activity with increasing peak grip force (right hand) in left ventrolateral premotor cortex. We were therefore interested to examine post hoc for age-related changes in the relationship between ventrolateral premotor cortex activity and peak grip force. Regions of interest were created as follows. We created a region of interest using a 20 mm diameter sphere centred on the coordinates *x* = 48, *y* = 14, *z* = 16, and *x* = −42, *y* = 20, *z* = 18, derived from Ward and Frackowiak (2003). We then looked for voxels within each region of interest which exhibited age-related changes in *B*_F_ at a threshold of *P* < 0.001, uncorrected. With this approach, positive correlations between *B*_F_ and age^2^ were seen in ventrolateral premotor cortex/BA44 bilaterally for both right hand use (*x* = 50, *y* = 10, *z* = 18, *Z*-score = 3.11, *r*^2^ = 0.27 and *x* = −50, *y* = 20, *z* = 24, *Z*-score = 3.04, *r*^2^ = 0.29) and left hand use (*x* = 46, *y* = 22, *z* = 24, *Z*-score = 3.18, *r*^2^ = 0.26 and *x* = −46, *y* = 22, *z* = 34, *Z*-score = 3.37, *r*^2^ = 0.30) (Fig. 5). These results are presented as subthreshold trends and so are not reported in Table 5.

None of the above results were influenced by adding gender as an additional covariate in keeping with a previous absence of gender effect using the same hand grip paradigm (Ward and Frackowiak, 2003).

## 4 Discussion

We have used fMRI to study age-related changes in the extent and timing of motor system activation during a simple visuomotor task performed with each hand in a sparse event-related design. We have shown that the canonical HRF captures all the experimentally induced signal change in cortical regions involved in motor output and that the shape of the HRF in these regions varies little in relation to age. We have confirmed that there are increases but no decreases in motor system activation with increasing age, a process which is likely to accelerate as aging progresses. Lastly, by asking subjects to vary levels of force production we have been able to show for the first time that the motor cortices of older subjects are less able to increase activity when increasing force output is required. There is some evidence that increasing activity with greater force production is more prominent in the ventral premotor cortices of older compared to younger subjects.

### 4.1 Main effects of hand grip

The visuomotor task employed in this experiment activates a widely distributed brain network as previously reported (Ward and Frackowiak, 2003). The use of both temporal and dispersion derivatives of the canonical HRF has enabled the identification of regional variations in haemodynamic response during this task. Earlier responses were seen in the pulvinar bilaterally. The onset of our canonical HRF was taken as the beginning of hand grip rather than the prior visual cue, and thus earlier responses in the pulvinar are to be expected and are in keeping with its role in early selective visual attention (Robinson and McClurkin, 1989). Strong bilateral intraparietal sulcus activations were captured by the canonical HRF, but delayed responses captured by the temporal derivative were seen in right parietal structures centred upon the intraparietal sulcus and left cerebellum (crus I). These responses were lateralised in the brain independently of the hand used. The left parietal cortex is most often associated with attention towards a motor act (Rushworth et al., 2003, 2001), but increased activation in right parietal structures has been associated with the cessation of a motor act (Maguire et al., 2003; Rubia et al., 2001). Thus, sustained response in right parietal cortex might reflect cessation of hand grip, or possibly continued attention to visual feedback of force production after grip cessation. A similar delay in left cerebellar (crus I) activity irrespective of which hand was used may reflect parieto-cerebellar connections important in visually guided motor tasks (Brodal and Steen, 1983; Glickstein, 2003). Thus in this event-related study the synthetic canonical HRF, with onset at the beginning of hand grip, was able to account for almost all of the experimentally induced variance (Fig. 1). Variations in HRF were independent of the hand used, and so it is unlikely that these changes were directly related to the generation of lateralised motor output, but rather some cognitive aspect of the task common to both.

### 4.2 Age-related changes

A reduced BOLD signal to noise ratio (SNR) in M1 has been reported in older compared to younger subjects (D’Esposito et al., 1999). Differential SNR across ages is a potential problem when looking for age-related changes if the error variance is dominated by *within subject* variability, as in a fixed effects analysis. In this case the lower SNR will result in fewer suprathreshold voxels even though the magnitude of activation is no different (D’Esposito et al., 1999). The problem can be overcome by employing a random effects analysis where the error variance is dominated by *between subjects* variability, as in the current study. Thus, any age-related changes in magnitude of activation in this study are unlikely to be related to differential SNR.

We were particularly interested in age-related changes in M1, as previous studies have provided conflicting results (see Ward, 2006 for review). With regard to contralateral M1, there have been reports of decreases in extent (D’Esposito et al., 1999) or magnitude (Hesselmann et al., 2001; Hutchinson et al., 2002; Riecker et al., 2006; Tekes et al., 2005; Wu and Hallett, 2005) of activation in older subjects, whereas others have reported no change (Calautti et al., 2001; Heuninckx et al., 2005; Ward and Frackowiak, 2003). Mattay et al. (2002) found increased contralateral M1 activity in older subjects, but only in those with a similar level of motor performance to the young subjects. The differences in results are likely to be due to the use of different paradigms ranging from simple to more complex motor tasks, and different methods of analysis which are more or less susceptible to the effects of reduced SNR in older subjects (D’Esposito et al., 1999). In our event-related study, we found no age-related changes in the average magnitude or shape of the BOLD response in contralateral M1, in keeping with the findings of D’Esposito et al. (1999).

More consistent changes have been found in ipsilateral M1. During motor tasks, there is a reduction in M1 BOLD signal ipsilateral to the moving hand (Allison et al., 2000; Newton et al., 2005), but in older subjects, this deactivation appears reduced (Hutchinson et al., 2002; Naccarato et al., 2006; Riecker et al., 2006; Ward and Frackowiak, 2003). In a previous study employing hand grip, we found decreasing ipsilateral M1 deactivation with increasing age^2^ (Ward and Frackowiak, 2003) for both left and right hand use. We have replicated this result, which supports the recent finding of a shift in laterality towards ipsilateral M1 with increasing age during a finger opposition task (Naccarato et al., 2006). The mechanism of deactivation in ipsilateral M1 during the performance of a motor task is thought to be via transcallosal inhibition. Older subjects appear to have reduced excitability of intracortical inhibitory circuits in motor cortex as assessed with short interval paired pulse TMS (Kossev et al., 2002; Peinemann et al., 2001) and the EMG silent period (Eisen et al., 1996; Prout and Eisen, 1994; Sale and Semmler, 2005). It is therefore possible that aging also leads to impaired transcallosal inhibition of ipsilateral M1. However, it is interesting to speculate that our finding of age-related increases in task-related signal in the putamen bilaterally suggests that changes cortico-subcortical connections may also play a role in this shift in hemispheric balance, as M1 and putamen are intimately connected (Kelly and Strick, 2004). Mirror movements are often considered to confound the interpretation of ipsilateral M1 activation. We did not observe mirror movements during task practice outside the scanner in any subject, and neither did we detect mirror gripping during scanning with our force transducers. However, we cannot rule out a very small level of mirror activity not picked up by our methods, which might be detectable only by careful EMG. However, we would suggest that any mirror EMG activity is likely to be the product of a less inhibited ipsilateral M1 rather than confounding voluntary activity.

As well as changes in M1 recruitment, age-related changes have also been reported in non-M1 regions during the performance of a variety of motor tasks (Heuninckx et al., 2005; Hutchinson et al., 2002; Mattay et al., 2002; Rowe et al., 2006; Ward and Frackowiak, 2003; Wu and Hallett, 2005). Increased recruitment in non-motor brain regions with age is more prominent in complex motor tasks (Heuninckx et al., 2005) suggesting increased cognitive monitoring of performance. In the current study, increasing task-related activity in bilateral dorsolateral premotor cortex, left intraparietal cortex, and right cerebellum (lobule VI) were observed with increasing age with left but not right hand use. This suggests that increased monitoring or attention to the performance of the non-dominant left hand is required in the older subjects.

The inclusion of temporal and dispersion derivatives allowed us to look for systematic age-related changes in the shape or timing of the haemodynamic response. Aging can affect neurovascular coupling and therefore the form of the haemodynamic response. Taoka et al. (1998) reported a slower task-related rise in M1 BOLD signal in older subjects, whereas others have reported no difference in the shape of the M1 haemodynamic response (D’Esposito et al., 1999). Most subsequent studies examining age-related effects have employed blocked designs which, although efficient, preclude examination of haemodynamic response variations. Using a sparse event-related design, we found no age-related changes in the shape of haemodynamic response in M1 for this task. However, we did find that increasing age is associated with prolonged haemodynamic responses in bilateral intraparietal sulcus and cerebellum when the left hand is used. Such changes were limited to right intraparietal sulcus for right hand use. Although this could be the result of an altered neurovascular response in these brain regions, the result would also be in keeping with increased and/or prolonged attention to the motor task and monitoring for errors in older subjects, especially when the less automatic non-dominant left hand is used.

If regional changes in neurovascular coupling were occurring as a function of age, then a similar relationship between age and the temporal and dispersion derivatives should have been seen for both right and left hand scanning sessions, since much of the activation outside M1 was bilateral. This was not the case, and it is therefore highly likely that the regional alterations in haemodynamic shape and timing are attributable to differences in cognitive approaches to the tasks. This is an important finding which suggests that fMRI is an appropriate tool for studying age-related changes in motor-related cortical regions.

### 4.3 Force-related change

Parametric modulation of target forces allows us to examine how the cortical motor system responds when increasing force output is required. For the group as a whole we observed a positive correlation between BOLD signal and grip force in contralateral M1 and superior cingulate sulcus, ipsilateral cerebellum, and primary visual cortex (in response to greater visual stimulation/feedback with increasing force), replicating the findings of previous studies (Dettmers et al., 1995; Thickbroom et al., 1998; Ward and Frackowiak, 2003). In contralateral M1 and cingulate sulcus however, the correlation diminished with advancing age for both right and left hand tasks. A similar decline was seen in contralateral primary sensory cortex and PMd for the right hand. Thus, our fMRI data suggests that when increasing force output is required within the range of 15–45% of maximum hand grip, then cortical regions known to contribute to the corticospinal tract (Dum and Strick, 1991) are less able to increase output-related activity with advancing age. This novel finding is in keeping with results from TMS experiments which demonstrate that older subjects generally have lower MEP amplitudes in response to submaximal TMS (Eisen et al., 1991), whereas maximal MEP amplitudes are similar in all age groups (Pitcher et al., 2003). This age-related change in stimulus-output characteristics suggests that the number of large diameter corticospinal fibres does not decline appreciably with advancing age, but the ability to activate these fibres with TMS is reduced (Pitcher et al., 2003; Sale and Semmler, 2005). Furthermore, accelerated decline of grey matter volume with advancing age has been reported in central and cingulate sulci compared to other regions (Good et al., 2001). It is interesting to speculate that both the TMS and fMRI results reflect the functional consequences of these regionally specific effects. An alternative explanation for our results might arise from the finding that in older subjects there is an increased variability of motor unit discharge in response to increasing force output (Sosnoff et al., 2004; Tracy et al., 2005; Vaillancourt et al., 2003). If the relationship between brain and muscle activity is altered in older subjects, then increases in force production might be less reliably reflected in changing BOLD signal. Our current results cannot distinguish between these possibilities.

Despite these findings, all subjects were able to modulate their grip force. We found a weakly positive correlation between *B*_F_ and age^2^ in PMv bilaterally, replicating a previous finding (Ward and Frackowiak, 2003). In other words, a positive correlation between BOLD signal in PMv and force output was more likely in older subjects. A small proportion of PMv cells may either increase or decrease neuronal firing rates with increasing precision grip force (Hepp-Reymond et al., 1994), but our results demonstrate that a consistently positive correlation between force output and BOLD signal was more likely to be seen in PMv rather than M1 with advancing age. Premotor regions, particularly PMv, are more active during precision compared to power grip (Ehrsson et al., 2000, 2001). It is possible that older subjects were performing the force modulation task more like a precision grip task thus accounting for the trend towards increased modulatory behaviour in PMv. Nevertheless, it still suggests that PMv becomes increasingly functionally useful with increasing grip force with increasing age. In the older brain, existing inputs to M1 may be insufficient to increase output to spinal cord motor neurons when higher grip forces are required. In normal primates rostral PMv (area F5) is able to facilitate motor cortex output to upper limb motor neurons (Cerri et al., 2003; Shimazu et al., 2004). Thus, additional PMv input to M1 could exert a modulatory effect by increasing the gain of M1 output.

### 4.4 The rate of age-related change in the cortical motor system

Most previous studies have made categorical comparisons between old and young subjects. Naccarato et al. (2006) recently looked for a correlation between age and a measure of the shift in laterality from contralateral to ipsilateral M1 during a simple motor task. We have previously also used a correlational approach rather than categorical comparison to look for age-related changes in motor system activation (Ward and Frackowiak, 2003) although, based on the behavioural observations that decline in motor function is non-linear and accelerates beyond the age of 60 years (Smith et al., 1999), we used age^2^ rather than age as a covariate. We have used the same approach in the current study. Correlational approaches avoid making assumptions about what constitutes ‘old’ or ‘young’, and also acknowledges that the processes under investigation may be continuous throughout adult life

In summary, we have demonstrated that the configuration of the cortical motor system during a simple hand grip task changes with advancing age. Furthermore, it is clear that the way in which the motor system responds to the demands of increasing force production also changes with age. The reduced ability to modulate activity in appropriate motor networks when required may be a contributory factor in the decline of motor performance in older subjects. Furthermore, because of our experimental design, these results are likely to reflect structural and neurophysiological age-related changes in motor-related brain regions rather than purely changes in cognitive strategy.

## Conflict of interest

All authors confirm that they have no financial, actual or potential, conflicts of interest that could inappropriately influence or bias this work.

## Figures and Tables

**Table 1:** The neural correlates of temporal and dispersion derivatives

Region	Right hand					Left hand				
	Side	Talairach coordinates in MNI space			*Z*-value	Side	Talairach coordinates in MNI space			*Z*-value
		*x*	*y*	*z*			*x*	*y*	*z*	
Temporal derivative										
(i) Earlier responses										
Pulvinar	R	8	−24	−4	5.71	R	6	−20	−2	5.55
	L	−6	−22	−2	5.41	L	−4	−20	0	5.27

(ii) Later responses										
Superior parietal cortex	R	52	−36	56	6.61	R	40	−48	62	5.86
Intraparietal sulcus	R	40	−46	42	5.68	R	46	−40	42	5.48
Intraparietal sulcus	R	34	−72	46	5.59	R	28	−70	56	5.17
Inferior parietal cortex	R	60	−32	46	5.95	R	56	−40	48	6.48
Cerebellum (Cr I)	L	−26	−7	−28	5.92	L	−40	−76	−30	5.72
Cerebellum (Cr I)	L	−46	−68	−32	5.21	L	−44	−58	−46	5.69
Cerebellum (Cr I)						L	−38	−56	−34	5.54

Dispersion derivative										
(i) Greater dispersion										
None										

(ii) Less dispersion										
Superior cingulate sulcus	L	−8	−22	46	5.85	L	−8	−18	46	6.71

Voxels in which some part of the task-related response is accounted for by the temporal or dispersion derivatives of the canonical haemodynamic response function. All voxels are significant at *P* < 0.05, corrected for multiple comparisons across whole brain.

**Table 2:** Force-related changes

Region	Right hand					Left hand				
	Side	Talairach coordinates in MNI space			*Z*-value	Side	Talairach coordinates in MNI space			*Z*-value
		*x*	*y*	*z*			*x*	*y*	*z*	
(i) Increasing signal with increasing force										
Primary motor cortex (M1)	L	−36	−18	58	5.55	R	36	−20	58	4.93
	L	−40	−14	46	4.78					

Superior cingulate sulcus	L	−4	−16	50	4.87	R	6	−2	48	4.63

Cerebellum (lobule VI)	R	28	−44	−28	5.61	R	26	−60	−20	5.11
						L	−24	−56	−24	5.12

Primary visual cortex (V1)	L/R	0	−82	2	5.05	L/R	0	−92	6	5.89

(ii) Decreasing signal with increasing force										
None										

Voxels in which hand grip-related signal change increases linearly with increasing hand grip force. All voxels are significant at *P* < 0.05, corrected for multiple comparisons across whole brain.

**Table 3:** Age-related changes in the main effects of hand grip

Region	Right hand						Left hand					
	Side	Talairach coordinates in MNI space			*Z*-value	Correlation analysis (*r*^2^)	Side	Talairach coordinates in MNI space			*Z*-value	Correlation analysis (*r*^2^)
		*x*	*y*	*z*				*x*	*y*	*z*		
(i) Increasing signal with increasing age^2^												
Primary motor cortex (M1)	R	42	−18	54	4.36	0.40	L	−26	−20	58	4.78^*^	0.43
							L	−22	−22	66	4.76^*^	0.42

Dorsolateral premotor cortex (PMd)							R	26	−16	68	4.57	0.38
							L	−24	−8	56	4.56	0.46

Intraparietal sulcus							L	−34	−34	36	4.59	0.41
Putamen	R	28	−4	2	4.33	0.38	R	30	−8	6	4.47	0.38
	L	−26	−8	8	4.41	0.32	L	−30	−6	4	4.90^*^	0.55

Cerebellum (lobule VI)							R	16	−56	−20	4.41	0.46

(ii) Decreasing signal with increasing age^2^												
None												

Voxels in which there is a correlation between the magnitude of activation during hand grip (*B*_G_) and age^2^. All peak voxels are significant at a height threshold of *P* < 0.001, uncorrected, and extent (cluster) threshold of *P* < 0.05, corrected for multiple comparisons across whole brain. *Z*-values marked ‘*’ indicate that the voxel is significant at a height threshold of *P* < 0.05, corrected for multiple comparisons across whole brain.

**Table 4:** Age-related changes in dispersion derivatives

Region	Right hand						Left hand					
	Side	Talairach coordinates in MNI space			*Z*-value	Correlation analysis (*r*^2^)	Side	Talairach coordinates in MNI space			*Z*-value	Correlation analysis (*r*^2^)
		*x*	*y*	*z*				*x*	*y*	*z*		
Increasing dispersion with increasing age^2^												
Intraparietal sulcus	R	38	−62	38	4.80^*^	0.52	R	28	−72	48	4.15	0.39
							L	−22	−72	50	4.17	0.43

Cerebellum (lobule VI)							R	40	−48	−28	4.99^*^	0.57
							L	−48	−56	−28	4.98^*^	0.55

Decreasing dispersion with increasing age^2^												
None												

Voxels in which there is a correlation between the parameter estimate for the temporal (*B*_T_) or dispersion (*B*_D_) derivative of the haemodynamic response function during hand grip and age^2^. All peak voxels are significant at a height threshold of *P* < 0.001, uncorrected, and extent (cluster) threshold of *P* < 0.05, corrected for multiple comparisons across whole brain. Voxels marked ‘*’ are significant at a height threshold of *P* < 0.05, corrected for multiple comparisons across whole brain. Cerebellar localization performed from Schmahmann et al. (1999).

**Table 5:** Changing force modulation with age

Region	Right hand						Left hand					
	Side	Talairach coordinates in MNI space			*Z*-value	Correlation analysis (*r*^2^)	Side	Talairach coordinates in MNI space			*Z*-value	Correlation analysis (*r*^2^)
		*x*	*y*	*z*				*x*	*y*	*z*		
(i) Decreasing force modulation with increasing age^2^												
Primary sensory cortex (S1)	L	−48	−16	58	4.76^*^	0.39						
Primary motor cortex (M1)	L	−34	−26	58	4.21	0.37	R	28	−22	62	4.34	0.38
Dorsolateral premotor cortex (PMd)	L	−44	−12	58	4.34	0.45						
Superior cingulate sulcus	L	−12	−6	40	3.83	0.45	R	14	−26	48	3.76	0.41

(ii) Increasing force modulation with increasing age^2^												
None												

Voxels in which there is a correlation between the parameter estimate representing linear changes in BOLD signal with grip force (*B*_F_) during right hand grip and age^2^. All peak voxels are significant at a height threshold of *P* < 0.001, uncorrected, and extent (cluster) threshold of *P* < 0.05, corrected for multiple comparisons across whole brain. Voxels marked ‘*’ are significant at a height threshold of *P* < 0.05, corrected for multiple comparisons across whole brain.
